# Editorial: The Role of the Fungal Cell Wall in Host-Fungal Interactions

**DOI:** 10.3389/fcimb.2020.00392

**Published:** 2020-07-29

**Authors:** Laura Alcazar-Fuoli, Jagadeesh Bayry, Vishukumar Aimanianda

**Affiliations:** ^1^Mycology Reference Laboratory, National Centre for Microbiology, Instituto de Salud Carlos III, Madrid, Spain; ^2^Spanish Network for Research in Infectious Diseases, Instituto de Salud Carlos III, Madrid, Spain; ^3^Institut National de la Santé et de la Recherche Médicale, Centre de Recherché des Cordeliers, Sorbonne Université, Université de Paris, Paris, France; ^4^Institut Pasteur, Molecular Mycology Unit, CNRS, UMR2000, Paris, France

**Keywords:** fungal cell-wall, biosynthesis, remodeling, host-fungal interaction, antifungals

By providing mechanical strength and protection from the ever-changing hostile environment, the cell-wall (CW) forms an essential structure of fungal cells. Concerning host-pathogen interaction, the CW is the first fungal structure to interact with the host. It is a dynamic organelle with complex composition, varying between fungal species, morphotypes, and growth conditions, which poses difficulties in deciphering its role during host-fungal interactions. Therefore, new strategies to understand CW-organization are needed to improve the management of fungal infections. While CW-directed antifungals show good/acceptable efficacy, their clinical application is limited to echinocandins that inhibit biosynthesis of β-(1,3)-glucan, a major component in the fungal CW. Echinocandins are used for salvage therapy against invasive fungal infections (IFI) owing to their toxicity, paradoxical effect at higher doses, and due to the emergence of fungal resistance against echinocandins. This demands a necessity to discover alternative CW-targets and to develop new antifungals. On the other hand, in spite of medical advances, diagnostic-delay is attributed to be one of the reasons for increasing mortality due to IFI. Although circulating CW-antigens have been proven to be diagnostic biomarkers, the existing protocols suffer from specificity and sensitivity issues, requiring new tools overcoming these drawbacks. In our focused topic, the nine articles collected highlight recent developments regarding the fungal CW in these research areas.

A protective immune response relies on recognition of fungal-pathogens by pattern recognition molecules of the host immune-system. The review by Madan and Kishore summarizes the host immune surveillance role of the Surfactant Protein D (SP-D), a pattern-recognition receptor, in recognizing and eliminating human fungal pathogens. The CW-ligands interacting with SP-D, mechanism of interactions and immunomodulatory effects thereby are discussed. Fungicidal or fungistatic affect exerted by, and therapeutic potentials of SP-D upon external administration in murine models of allergic and invasive mycoses are highlighted.

Fungal-keratitis is a superficial infection mainly due to the species of *Aspergillus* and *Fusarium*; although not life-threatening, this infection greatly affects the quality of life. In the research article by Mohammed et al. local activation of alternative complement pathway, a humoral immune defense mechanism of the host during early stage of corneal-infection by *A. flavus*, has been demonstrated. They have also identified the negative regulators of complement activation, capable of interacting with *A. flavus*, demonstrating a parallel immune evasion mechanism associated with this fungus during corneal-infection.

Being an extracellular phenomenon, the fungal CW-biogenesis relies on a coordinated function of several glycosyltransferases; among them, β-(1,3)-glycosyltransferases of the GH75 family (CAZyme) play essential roles. The brief research-report by Degani and Popolo describes the role of Phr1p, a β-(1,3)-glycosyltransferases, in maintaining *Candida albicans* CW integrity, by acting cooperatively with a chitin synthase, Chs1p. Further, Phr1p-GFP construct allowed them to localize Phr1p in the septum of *C. albicans* undergoing cytokinesis, suggesting the utility of fluorescent protein tagging in fungal CW-biogenesis.

Chitin, although not a major component, maintains fungal CW-integrity upon cross-linking with β-glucans. Leroy et al. investigated the role of *C. albicans* CW-chitin released into the bloodstream during candidemia on platelets activity, as platelets are important during innate immune response. They observed that the chitin purified from *C. albicans* reduces adhesion of platelet to this fungus as well as neutrophils, thereby promoting fungal escape from immune cells. Pre-treatment of platelets with chitin resulted in their reduced aggregation by reducing intracellular Ca^+2^-influx and P-selectin expression in platelets, thus affecting platelet-leukocyte interaction and neutrophil recruitment to the sites of infection. This brings new insight into the pathobiological role of fungal CW-chitin. In some pathogenic fungi, chitosan, the deacetylated derivative of chitin also plays a role in virulence. However, Mouyna et al. demonstrate that although there are seven putative chitin deacetylases (Cda; converting chitin to chitosan) in *Aspergillus fumigatus*, an airborne pathogen, the chitosan level in *A. fumigatus* conidia (infective propagules) is very low. Further, deletion of all seven-Cda did not alter the growth and virulence, suggesting a non-essential role of CW-chitosan in the *A. fumigatus* biology/pathobiology. On the other hand, galactosaminogalactan, a heteropolysaccharide in the CW of *A. fumigatus*, produced during germination, exerts anti-inflammatory property upon inducing IL-1Ra by peripheral blood mononuclear cells. However, therapeutic application of this polymer is limited due to its acid-soluble nature. The research by Gressler et al. demonstrate that the oligosaccharides of galactosaminogalactan with 13–20 monosaccharide-units rich in de-*N*-acetylated galactosamine are water-soluble, capable of inducing IL-1Ra and can rescue inflammatory damage in colitis mouse model, suggesting the potential of CW-oligosaccharides as glycodrugs.

Infecting capacity and antifungal susceptibility varies across various species of *Candida*. Walker and Munro observed that caspofungin (an echinocandin) treatment results in reorganization of the CW in most *Candida* species (except *C. glabrata* and *C. parapsilosis*), exposing chitin and β-(1,3)-glucan (polysaccharides in the inner CW) that inhibited *Candida* uptake by macrophages, decreasing their TNF-α production. This study demonstrates drug-induced modifications in the CWs of *Candida* species, affecting their interaction with immune cells.

IFI occur mainly during immunosuppressed condition, immunomodulators are therefore receiving attention as antifungal therapy. In this context, Paulovičová et al. generated biotinylated manno-oligosaccharides that mimic CW-mannan of *Candida*, studied their immunomodulatory potential *in vitro*, which was dependent on the chain-lengths and linkage patterns of these oligoconjugates, thus suggesting their capacity as anti-*Candida* vaccines.

Galactomannan detection for the diagnosis of invasive aspergillosis suffers from false-positivity, due to cross-reactivity of the monoclonal antibodies (mAb) used, recognizing bacterial antigenic determinants. Using *A. parasiticus* CW-fragments as the immunogen, Schubert et al. developed AP3, a mouse mAb that specifically recognizes β-(1,5)-galactofuranose with a minimum length of tetramer, a structure common among many *Aspergillus* species. Owing to the higher epitope-specificity of AP3, its efficient application in invasive aspergillosis diagnosis has been envisaged.

Altogether, this themed article collection adds to our current knowledge on tools to study fungal CW-organization, immunomodulatory role of CW during host-fungal interaction, synthetic derivatives of CW in immunotherapies and CW-directed mAb in the diagnosis of fungal disease.

## Author Contributions

VA drafted the manuscript. All authors contributed to the revision and approved the submitted version.

## Conflict of Interest

The authors declare that the research was conducted in the absence of any commercial or financial relationships that could be construed as a potential conflict of interest.

